# Sigmoid colon cancer arising in a diverticulum of the colon with involvement of the urinary bladder: a case report and review of the literature

**DOI:** 10.1186/1471-230X-14-90

**Published:** 2014-05-13

**Authors:** Yasumichi Yagi, Yasuhiro Shoji, Shozo Sasaki, Akemi Yoshikawa, Yuji Tsukioka, Wataru Fukushima, Hisashi Hirosawa, Ryohei Izumi, Katsuhiko Saito

**Affiliations:** 1 Department of Surgery, Toyama City Hospital, 2-1 Imaizumi Hokubu-machi, Toyama 939-8511, Japan; 2 Department of Pathology, Toyama City Hospital, 2-1 Imaizumi Hokubu-machi, Toyama 939-8511, Japan

**Keywords:** Colon cancer, Diverticulum, Urinary bladder invasion

## Abstract

**Background:**

Colon cancer can arise from the mucosa in a colonic diverticulum. Although colon diverticulum is a common disease, few cases have been previously reported on colon cancer associated with a diverticulum. We report a rare case of sigmoid colon cancer arising in a diverticulum with involvement of the urinary bladder, which presented characteristic radiographic images.

**Case presentation:**

A 73-year-old man was admitted to our hospital for macroscopic hematuria. Computed tomography and magnetic resonance imaging revealed a sigmoid colon tumor that protruded into the urinary bladder lumen. The radiographs showed a tumor with a characteristic dumbbell-shaped appearance. Colonoscopy showed a type 1 cancer and multiple diverticula in the sigmoid colon. A diagnosis of sigmoid colon cancer with involvement of the urinary bladder was made based on the pathological findings of the biopsied specimens. We performed sigmoidectomy and total resection of the urinary bladder with colostomy and urinary tract diversion. Histopathological findings showed the presence of a colovesical fistula due to extramurally growing colon cancer. Around the colon cancer, the normal colon mucosa was depressed sharply with lack of the muscular layer, suggesting that the colon cancer was arising from a colon diverticulum.

**Conclusion:**

The present case is the first report of sigmoid colon cancer arising in a diverticulum with involvement of the urinary bladder. Due to an accurate preoperative radiological diagnosis, we were able to successfully perform a curative resection for sigmoid colon cancer arising in a diverticulum with involvement of the urinary bladder.

## Background

Colon cancer can arise from the mucosa in a colonic diverticulum. Although colon diverticulum is a common disease, few cases have been previously reported on colon cancer associated with a diverticulum [1-8]. Because a diverticulum lacks the muscular layer, cancerous tissue arising within a diverticulum can easily penetrate the serosa as it grows and may not be detected until an advanced stage [4-6]. Moreover, the specific progression with intramural growth may make it difficult to exactly diagnose. Herein, we report a rare case of sigmoid colon cancer arising in a diverticulum with involvement of the urinary bladder, which was accurately diagnosed by radiological images.

## Case presentation

**Figure 1:**
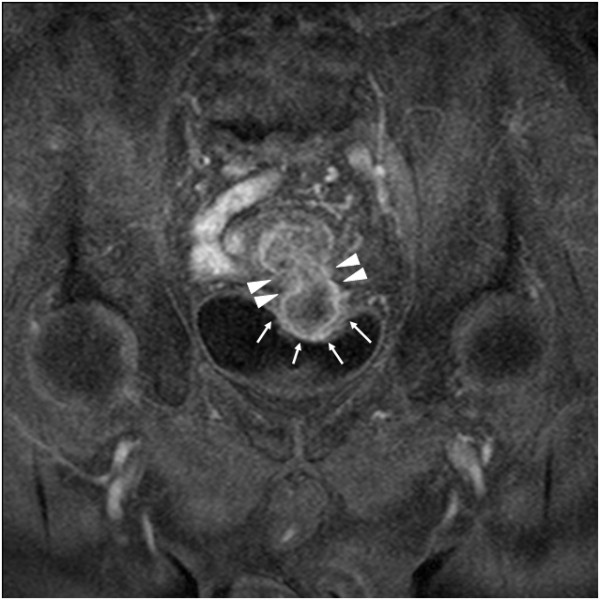
**Enhanced MRI T1-weighted image.** Between the sigmoid colon and the urinary bladder, the solid tumor was connected via the fistula, showing a dumbbell-shaped appearance. The sigmoid colon tumor protruded into the urinary bladder lumen (arrows) via the colovescal fistula (arrowheads).

**Figure 2:**
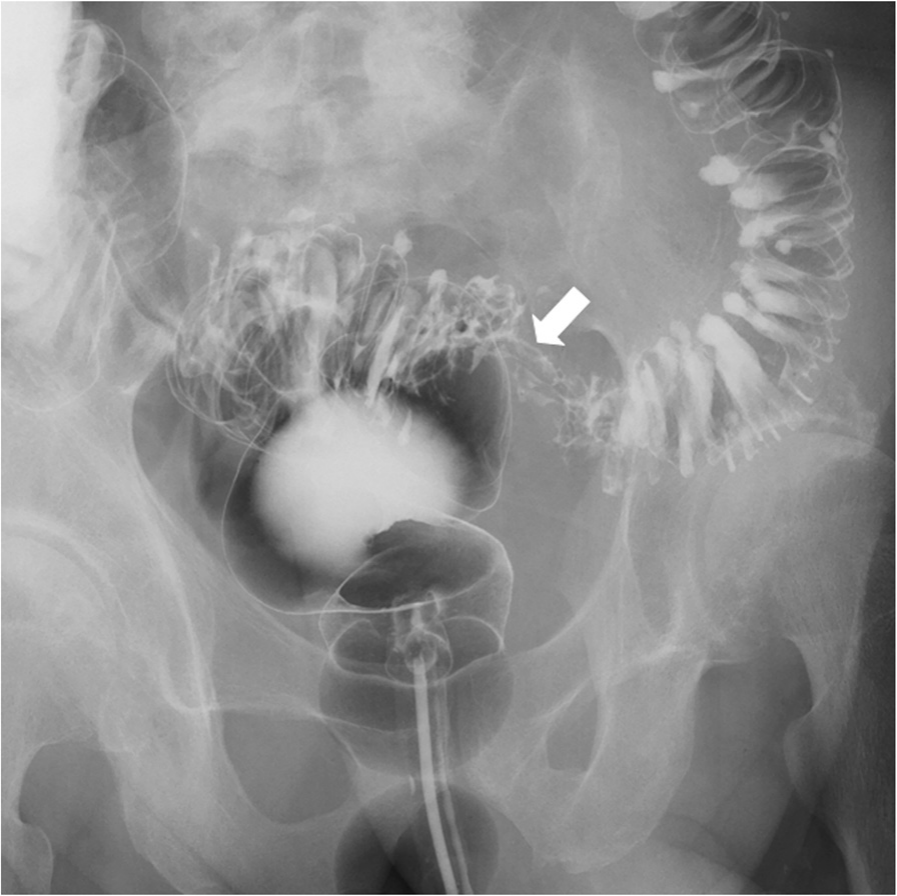
**Barium enema findings.** The colon tumor was detected as a defect in the sigmoid colon (arrow). Around the sigmoid colon cancer, there were multiple colon diverticula in the sigmoid colon.

**Figure 3:**
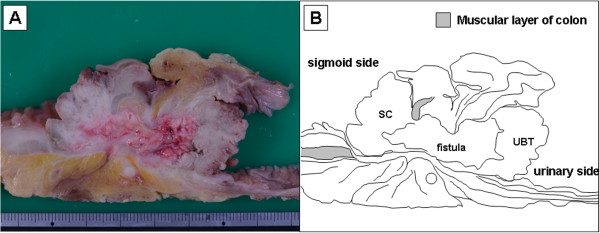
**Cross section of the resected specimen. A:** Macroscopic picture of a cross section. **B:** Diagram of a cross section. The progression of the sigmoid colon cancer was reflected in the MRI findings. The macroscopic findings revealed the progression via the colovesical fistula. SC: sigmoid colon cancer. UBT: urinary bladder tumor.

**Figure 4:**
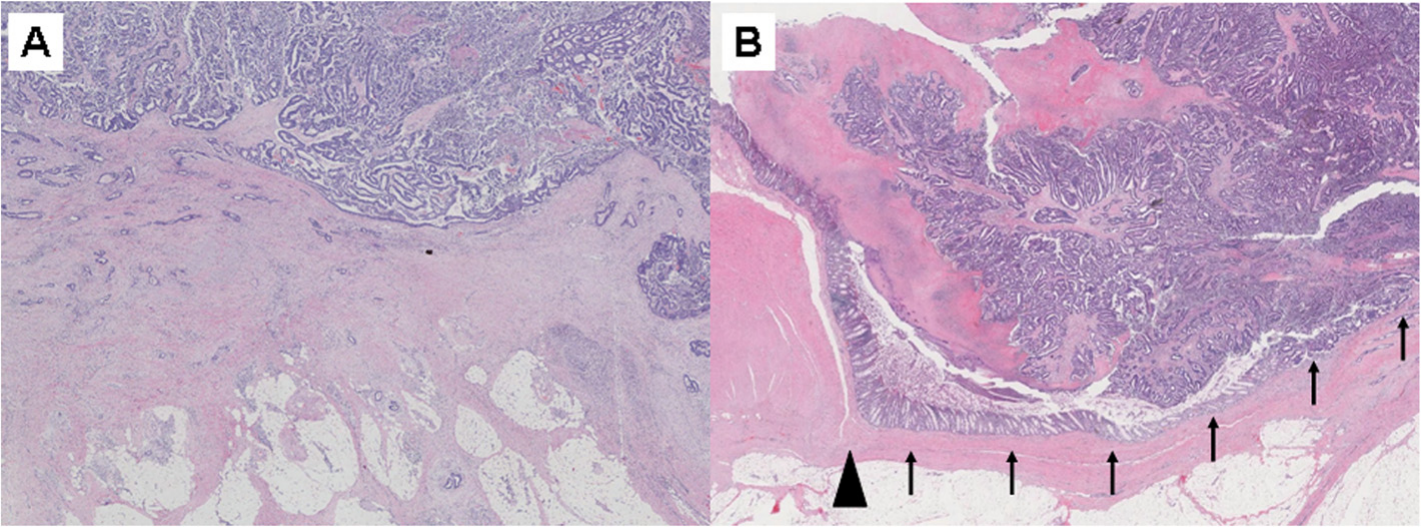
**Microscopic findings of the resected specimen (H-E staining, ×200). A:** The sigmoid colon cancer, penetrating to the urinary bladder, did not invade the surrounding muscular layer as the tumor progressed. Adjacent to the sigmoid tumor, the normal colon mucosa was depressed sharply (arrows) with a thin-walled muscular layer (arrowhead), indicating that the colon cancer had arisen from a colon diverticulum. **B:** The sigmoid and urinary sides of the tumor were connected via the colovesical fistula. In the fistula, the cancerous tissue was completely covered with fibrous tissue, indicative of no exposure to the intraperitoneal space to cancerous tissue.

A 73-year-old man was admitted to our hospital for macroscopic hematuria. Enhanced abdominal computed tomography (CT) revealed a sigmoid colon tumor which protruded into the urinary bladder lumen. Neither nodal metastasis nor distant metastasis was detected. Enhanced abdominal magnetic resonance imaging (MRI) also revealed a solid tumor in the sigmoid colon and the urinary bladder, both of which were connected to each other by a fistula. Both the CT and the MRI presented a characteristic dumbbell-shaped appearance (Figure 1). Colonoscopy showed a type 1 tumor and multiple diverticula in the sigmoid colon. The pathological diagnosis of the biopsied sigmoid colon tumor was well-differentiated tubular adenocarcinoma. Barium enema examination showed a tumor and multiple diverticula in the sigmoid colon, but could not clarify a communication with the urinary bladder lumen (Figure 2). Cystoscopic examination showed a tumor in the urinary bladder lumen. The pathological diagnosis of the biopsied bladder tumor was well-differentiated tubular adenocarcinoma, not transitional cell cancer, deriving from the sigmoid colon cancer. The laboratory data, including inflammatory reaction and tumor markers, were within normal limits. On the other hand, occult hematuria was detected due to the urinary bladder tumor. Thereafter, the diagnosis was established as sigmoid colon cancer with involvement of the urinary bladder (cT4N0M0, cStageIIB according to the UICC TNM classification). The therapeutic strategy was explained to the patient, who decided to undergo a surgical resection. We performed sigmoidectomy and total resection of the urinary bladder with colostomy and urinary tract diversion. The postoperative period was uneventful. Pathological examination of the resected specimen revealed well-differentiated tubular adenocarcinoma without nodal metastasis of the regional lymph node (pT4N0M0, pStageIIB), confirming the preoperative diagnosis. Macroscopic cross section of the resected specimen exhibited a dumbbell-shaped appearance, just as was shown in the MRI findings (Figure 3). Histopathological findings showed the presence of a colovesical fistula due to extramurally growing colon cancer. However, the intraperitoneal space was not exposed to cancerous tissue because the colovesical fistula was completely covered with a fibrous tissue layer (Figure 4A). In addition, around the colon cancer, the normal colon mucosa was depressed sharply with a thin-walled muscular layer, suggesting the presence of a colon diverticulum. The cancerous tissue protruded from the base of the inverted mucosa of the diverticulum to the colovesical fistula. The surrounding muscular layer was not involved in the process of the tumor progression. Thereafter, it was demonstrated that the colon cancer had arisen from the mucosa of the colon diverticulum (Figure 4B). The unique radiological findings of the present case were based on the histological findings of the progression via the colovesical fistula derived from a colon diverticulum. No evidence of recurrence was found at 12 months follow-up.

## Discussion

Cancer may incidentally arise in a colonic diverticulum. Based on an endoscopic finding of a tumor within a diverticulum, a diagnosis of early colon cancer arising in a diverticulum can be made [3,5,8]. In regard to cases of advanced colon cancer, the diagnosis mostly depends on the histological findings of the resected specimen, except for a tumor arising in a large diverticulum. However, diagnosis may be complicated by abnormal findings, such as abscess formation, submucosal progression, and diverticulitis [2-4]. In the present case, the pathological examination exhibited characteristic findings that the primary tumor of the sigmoid colon progressed via the colovesical fistula without peritoneal exposure. Moreover, the fistula was continuous with the inverted colon mucosa of the diverticulum. On the basis of these microscopic findings, the sigmoid colon cancer progressed along the structure of the fistula which was supposed to arise from the diverticulitis of the sigmoid colon since before the genesis of the sigmoid colon cancer. The irregular progression was plainly reflected in the radiological finding of a dumbbell-shaped tumor.

The sigmoid colon and rectum are common sites of a primary tumor invading the urinary bladder compared with other colon segments [9,10]. Previous reports have demonstrated this; even in cases of local advanced colorectal cancer with a colovesical fistula, extended surgery with en-bloc bladder resection contributes to local control and improvement of survival [11,12]. In short, the prognosis depends on the negative surgical margin and the status of the nodal metastasis with or without a colovesical fistula [13,14]. However, the extended resection requires partial or total resection of the bladder with urinary tract diversion. In the present case, it was inadequate to preserve the bladder, because the tumor invaded the triangle of the bladder. If the tumor was located apart from the triangle of the bladder, it should be considered to preserve the bladder with the partial resection of the bladder. Thereafter, precise radiological diagnosis preoperatively is recommended in order to properly determine the extent of the surgical resection.

According to previous reports, there have been ten cases of colon cancer arising in a diverticulum [1-8]. Of these ten cases, two were early cancer, and the other eight were advanced stage. As for the location of the lesion, in three cases it was the right side of the colon, and in the other seven it was the left side of the colon. In two cases, the cancer was associated with diverticulitis. In the present case, it was not clear whether the cancer was associated with diverticulitis or fistula due to diverticulitis. From the patient’s history, the communication between the colon and the bladder lumen might be temporally formed with inflammation of the bladder. However, the fistula might not be necessarily certified at this advanced stage after the tumor occupied the lumen of both the colon and the bladder. To the best of our knowledge, the present case is the first report of sigmoid colon cancer arising from a diverticulum with involvement of the urinary bladder.

## Conclusion

In conclusion, due to an accurate preoperative radiological diagnosis, we were able to successfully perform a curative resection for sigmoid colon cancer arising in a diverticulum with involvement of the urinary bladder.

## Consent

Written informed consent was obtained from the patient for publication of this case report and any accompanying images. A copy of the written consent is available for review by the Editor-in-Chief of this journal.

## Competing interests

The authors declare that they have no competing interests in this paper.

## Authors’ contributions

YY edited the manuscript. YS, SS, AY, YT, WF, HH, and RI supervised the whole study. KS provided advice on the pathological findings. All authors read and approved the final manuscript.

## Pre-publication history

The pre-publication history for this paper can be accessed here:

http://www.biomedcentral.com/1471-230X/14/90/prepub
